# Sex differences in the capacity of minor phytocannabinoids to attenuate nociceptive insults in HIV-1 Tat-expressing mice

**DOI:** 10.1515/nipt-2024-0025

**Published:** 2025-07-17

**Authors:** Alaa N. Qrareya, Emaya Moss, Fakhri Mahdi, Mohammad F. Salahuddin, Duoyi Hu, Miguel A. De Leon, Amira S. Wanas, Mohamed M. Radwan, Mahmoud A. ElSohly, Nicole M. Ashpole, Jason J. Paris

**Affiliations:** Department of BioMolecular Sciences, University of Mississippi, University, MS, 38677, USA; Department of Pharmaceutical Sciences, School of Pharmacy, Notre Dame of Maryland University, Baltimore, MD, 21210, USA; National Center for Natural Products Research, University of Mississippi, University, MS, 38677, USA; Department of Pharmaceutics and Drug Delivery, University of Mississippi, University, MS, 38677, USA; Research Institute of Pharmaceutical Sciences, University of Mississippi, University, MS, 38677, USA

**Keywords:** behavioral pharmacology, cannabinoids, pain, sex differences, trans-activating transcriptor

## Abstract

**Objecives:**

Approximately 80 % of people living with HIV (PLWH) develop chronic pain and preclinical studies support the involvement of the HIV-1 regulatory protein, trans-activator of transcription (Tat). Phytocannabinoids may attenuate pain in PLWH; however, these data are controversial, and the biological mechanisms are difficult to untangle from psychosocial factors in people.

**Methods:**

We have examined the therapeutic capacity of minor phytocannabinoids to attenuate Tat-promoted visceral hyperalgesia (acetic acid writhing assay) and reflexive nociception (warm water tail flick assay) in transgenic mice. We hypothesized that conditional expression of Tat_1-86_ in male and female mice [Tat(+) mice] would amplify pain responses compared to controls [Tat(−) mice], and that phytocannabinoids could ameliorate these effects.

**Results:**

Irrespective of sex, Tat(+) mice demonstrated greater visceral pain responses than did Tat(−) controls. The phytocannabinoids, cannabigerolic acid (CBGA), cannabidiol (CBD), and cannabinol (CBN), attenuated Tat-induced visceral pain in both males and females. However, the effectiveness of these cannabinoids varied by sex with CBN being more efficacious in males, while cannabigerol (CBG) alleviated visceral pain only in Tat(+) females. Cannabidiolic acid (CBDA) and cannabidivarin (CBDV) were not effective in either sex. CBGA and CBG were also efficacious in the tail flick test among Tat(−) males and females, but demonstrated only small, sex-dependent effects to reverse Tat-induced nociception. CBD and CBN exerted little-to-no efficacy in this test.

**Conclusions:**

These data suggest that phytocannabinoids exert analgesia for HIV-related pain, potentially aiding in the development of personalized pain management strategies.

## Introduction

In the era of combined antiretroviral therapy (cART), chronic pain is a common condition experienced by people living with HIV (PLWH) [1], 2]. Up to 80 % of PLWH have experienced chronic pain in their lifetime [3], [4], [5], [6]. Such prevalence is greater than that reported in the seronegative population (∼20 %) [7], [8], [9]. Chronic pain results in functional impairments, increased risk of substance use disorder, and poorer medication adherence among PLWH [10], [11], [12]. Although the underlying etiology of HIV-related pain is poorly understood, both cytotoxic HIV proteins and older-generation antiretroviral therapies are demonstrated to contribute. For the latter, the cytotoxic effects of certain nucleoside reverse transcriptase inhibitors (including stavudine, didanosine and zalcitabine) are known to promote HIV-associated pain [13], [14], [15]. However, even with modern antiretrovirals, pain persists as a chief complaint among PLWH [16] implicating the contribution of neurotoxic viral proteins. Despite peripheral HIV suppression, cART cannot eradicate HIV from the central nervous system (CNS) and cannot prevent the production of certain HIV virotoxins, including the trans-activator of transcription (Tat) [17], 18]. In animal models, Tat is sufficient to induce and promote the progression of multiple pain modalities, perhaps via mechanisms of direct neural damage and indirect pro-inflammatory processes [19], [20], [21], [22], [23].

In the CNS, HIV-1 Tat is predominantly secreted from infected myeloid cells, including monocyte-derived cells (microglia/perivascular macrophages in the brain and microglia/monocyte-derived macrophages in the spinal cord) [24], 25]. A low production of CD4^+/^CD8^+^ T-cells cross into cerebrospinal fluid (CSF) or brain parenchyma [26], 27]. In transgenic mice, the conditional expression of Tat protein in the CNS is associated with a neuropathic-like response, including mechanical and cold allodynia [21], [22], [23]. These observations co-occurred with the activation of dorsal root ganglion (DRG) microglia/macrophages, Wallerian regression of epidermal nerve fibers [22], 23], impairment of nerve conductance [22], 28], and reduced expression of electron transport chain complex proteins and increased expression mitochondrial fission markers in sciatic nerve [28]. In cultured DRG neurons, Tat induces apoptosis, hyperexcitability, and axonal retraction [14]. As such, Tat may be a key contributor to painful neuropathies in PLWH via multiple mechanisms and therefore an ideal target for therapeutic intervention.

Traditional pain interventions, including opioids, anticonvulsants, and nonsteroidal anti-inflammatory drugs, have been used for pain management among PLWH [29], 30]. However, such interventions have limited analgesic efficacy [31], 32]. Opioids are prescribed to PLWH with untoward effects that raise the risk of substance abuse and mental health disorders [33], 34]. As such, alternative interventions are needed. Delta-9-tetrahydrocannabinol (Δ^9^-THC) and cannabidiol (CBD) exert efficacy alleviating chronic pain associated with fibromyalgia, diabetic neuropathy, and multiple sclerosis [35], [36], [37]. However, the psychoactive effects of Δ^9^-THC raise concerns for substance abuse [38]. These effects are thought to be the results of pharmacodynamic actions at cannabinoid receptor type 1 (CB1) within the CNS [39]. Thus, non-psychoactive minor phytocannabinoids, such as CBD, warrant investigation. This study aims to examine the analgesic capacity of non-psychoactive cannabinoids, including cannabigerolic acid (CBGA), cannabidiolic acid (CBDA), cannabidivarin (CBDV), CBD, cannabigerol (CBG) and cannabinol (CBN) utilizing Tat transgenic mice.

Herein, we assessed the contribution of Tat expression in the pathology of visceral inflammatory nociception and hyperalgesia as measured by an acetic acid writhing assay and a spinal reflex assay, respectively. We further assessed the capacity of non-psychoactive minor phytocannabinoids to attenuate these effects. We hypothesized that HIV-1 *tat* transgene expression in adult male and female mice would amplify pain modalities, such as inflammatory and thermal responses, which would be ameliorated by minor phytocannabinoids.

## Materials and methods

All procedures involving animals were preapproved by the Institutional Animal Care and Use Committee at the University of Mississippi and were conducted in accordance with the National Institutes of Health “Guide for the Care and Use of Laboratory Animals” (NIH Publication No. 85-23).

### Chemicals

**Figure 1:**
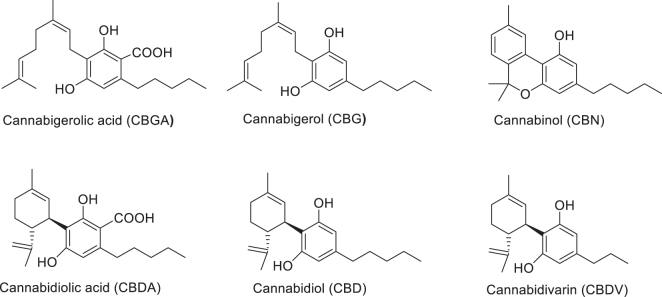
Chemical structures of non-psychoactive cannabinoid compounds used in the present study. Cannabigerolic acid (CBGA), cannabigerol (CBG), cannabinol (CBN), cannabidiolic acid (CBDA), cannabidiol (CBD), and cannabidivarin (CBDV).

Minor phytocannabinoids, including CBGA, CBDA, CBDV, CBD, CBG, and CBN (Figure 1) were obtained in-house from the National Center for Natural Product Research - Marijuana Research Laboratory (University, MS). Oxycodone was purchased from (Sigma-Aldrich, St. Louis, MO). Doxycycline was purchased from (Cayman Chemical, Ann Arbor, MI).

### Behavioral experiments: subjects and housing

HIV-1 Tat-transgenic mice were bred in the vivarium at the University of Mississippi (University, MS, USA). Mice were housed 2–5 per cage and kept in a temperature- and humidity-controlled environment on a 12:12 h light:dark cycle (lights off at 09:00 am) with *ad libitum* access to food and water. HIV-1 Tat_1–86_ is conditionally expressed by administration of doxycycline prepared fresh daily (30 mg/kg; Cayman Chemical, Ann Arbor, MI) injected subcutaneously (Acetic acid writhing assay) or intraperitoneally (Tail flick assay) for five consecutive days, followed by a 2-day wash-out period as described previously [40] to rule out non-specific effects of doxycycline (doxycycline _t1/2_ = 5–6 h; [41]. The use of a subcutaneous route of doxycycline administration prior to the acetic acid writhing assay prevents any confounding habituation to an intraperitoneal injection prior to testing. Briefly, Tat(+) mice express a *tat* transgene which is driven by a glial fibrillary acidic protein (GFAP), Tet-on promoter that becomes transcriptionally-active in the presence of a doxycycline-sensitive reverse tetracycline-controlled transactivating (rtTA) transcription factor. Tat(−) counterparts express the rtTA transcription factor but lack the *tat* transgene.

### Determination of estrous cycle phase

Estrous cycles were tracked by the daily collection of vaginal epithelia as previously described [42] with some modifications. Samples were collected at the beginning of the dark phase of the daily light cycle (∼09:00–10:00 h) and were evaluated. Estrous phases were identified based on epithelial cell morphology and the presence of leukocytes such that proestrus (predominance of nucleated cells), estrous (predominance of cornified cells), metestrus (presence of nucleated, cornified, and leukocytic cells), and diestrus (predominance of leukocytes) were determined [42]. Under the present conditions, the proestrus phase corresponds to the cycle phase wherein estradiol levels have peaked, and progestogens are rising to peak. These changes in circulating sex hormones occur concurrent with ovulation.

### Anti-nociceptive assessment

The assessment of nociceptive responses has been found to be influenced by hormonal fluctuations across the estrous cycle [43]. Female rodents in the proestrous phase of their estrous cycle exhibit greater anti-nociceptive behavior than males or females in the diestrous phase [44]. Estrogen may partly influence anti-nociceptive responding [45]. Consequently, female mice were tested during the proestrous phase of their estrous cycle. Females with irregular estrous cycles (no proestrous phase detected within 2 weeks) were excluded from the study. Male and female mice were acclimated to the behavioral testing room for 30 min prior to testing and were assessed approximately 1–3 h into the dark phase of their light cycle.

### Acetic acid-induced writhing test

Acetic acid-induced writhing was used to assess the analgesic capacity of agents as described [46], 47] with some modifications. Briefly, mice were intraperitoneally injected with vehicle (1:1:18 ethanol: kolliphor: saline) or minor phytocannabinoids (10 mg/kg). Thirty minutes later, 0.7 % acetic acid was injected into the intraperitoneal cavity on the opposing side (in a volume of 1 ml/0.1 kg) to induce an inflammatory response and activate nociceptors [48]. Mice were immediately placed in plexiglass observation chambers and abdominal stretching behavior was video-recorded for 30 min. Investigators that were blind to genotype and treatment conditions analyzed the number of stretches within 30 min. A greater frequency of stretching behavior indicates greater visceral pain.

### Tail flick (warm water tail withdrawal assay)

The tail flick assay was used to assess the spinal reflex response in mice as described [49] with some modifications. Briefly, a water bath was warmed and maintained at 56 °C. Mice were gently constrained with a soft tissue that exposed the tail. Approximately 1 cm of the tail was immersed in the water bath. The latency to tail withdrawal (rapid flick) was recorded with a 10 s cut-off (to avoid tissue damage). Prior to drug administration, the latency to tail withdrawal at baseline was measured. Afterward, a cumulative dosing curve (1.25, 2.5, 5, 10, and 20 mg/kg) of minor phytocannabinoids (CBGA, CBG, CBD, and CBN), vehicle (1:1:18 ethanol: kolliphor: saline), negative control (0.9 % saline), or positive control (oxycodone) was tested with a 30 min inter-trial interval (*see* Supplementary Table 1S *for oxycodone and corresponding vehicle performance*). For each dose, the mean of two consecutive measurements of tail flick were used. The analgesic effect of minor phytocannabinoids was assessed by converting the tail flick latency to percentage of Maximum Possible Effect (%MPE) using the following formula: %100 × (tail withdrawal latency post drug-baseline tail withdrawal)/cut-off tail withdrawal latency – baseline tail withdrawal latency). The %MPE was calculated for each treatment condition using at least six mice per group. The data presented as %MPE serves as a relevant comparison of oxycodone-mediated anti-nociception.

### Statistical analyses

Data were analyzed for each sex separately using repeated measures analyses of variance (ANOVAs) with Tat genotype [Tat(+) and Tat(−)] and drug condition [vehicle, CBGA, CBD, CBG, and CBN] as between-subjects factors and time or concentration as within-subjects factors. Group differences following main effects were delineated via Fisher’s Protected Least Significant Difference *post hoc* tests. Interactions were delineated via the assessment of simple main effects and main effect contrasts with alpha controlled for family-wise error. All data were considered significant when p ≤ 0.05. Outliers were determined by Dixon’s test and were excluded. Median effective doses (ED_50_; reported with 95 % confidence intervals) were determined via non-linear regression (sigmoidal curvilinear modeling with variable slope) using a least-squares fit for each treatment group (bottom and top values constrained to 0 and 100, respectively). All analyses were performed using SAS StatView or GraphPad Prism software.

## Results

### Minor phytocannabinoids attenuate Tat-induced visceral pain in male and/or female mice

**Figure 2:**
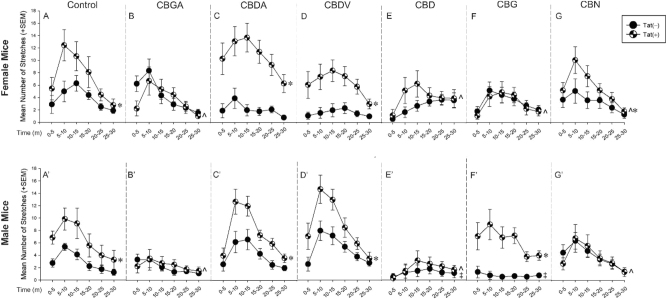
Visceral analgesic capacity of minor phytocannabinoids in HIV-1 Tat-tg mice. Female and male mice (*n* = 6–12/group) that conditionally-expressed Tat [Tat(+)] or their non-Tat expressing counterparts [Tat(−)] were assessed over 30 min in an acetic-acid writhing test. Mice were pretreated with vehicle or minor phytocannabinoids (10 mg/kg) 30 min before an intraperitoneal acetic acid (0.7 %) insult. Treatments included: (A,A′) vehicle control, (B,B′) CBGA, (C,C′) CBDA, (D,D′) CBDV, (E,E′) CBD, (F,F′) CBG, or (G,G′) CBN. *Indicates an interaction for Tat(+) mice to differ from control Tat(−) mice. ‡Indicates an interaction for Tat(−) mice to differ from respective control Tat(−) vehicle-treated mice. ^indicates an interaction for Tat(+) mice to differ from vehicle-treated Tat(+) mice (two-way repeated measures ANOVA, p≤0.05).

The analgesic capacity of minor phytocannabinoids at attenuating visceral pain was assessed in Tat-transgenic mice utilizing the acetic acid writhing test. Data were analyzed using two-way repeated measures ANOVAs with Tat genotype [Tat(+) and Tat(−)] and drug condition [vehicle, CBGA, CBDA, CBDV, CBD, CBG and CBN] as factors for each sex. There was an interaction between genotype [Tat(+) and Tat(−)] and drug condition in both females [*F*(6,590) = 4.92, p < 0.05; Figure 2] and males [*F*(6,595) = 2.88, p < 0.05; Figure 2]. Compared to Tat(−) vehicle-treated mice, Tat expression exacerbated visceral pain characterized by an increase in writhes among vehicle-treated females (p < 0.0001; Figure 2A see*) and males (p < 0.0001; Figure 2A’ see*). CBGA, CBD, and CBN attenuated Tat-mediated visceral pain in females (p < 0.0001; Figure 2B, E, and G see^) and males (p < 0.0001; Figure 2B’, E’, and G’ see^) compared to their control Tat(+) vehicle-treated mice, as indicated by the half-filled circles (Figure 2A, A’). However, CBDA and CBDV treatment worsened Tat-mediated visceral pain in females (p < 0.0001; Figure 2C and D see*) and males (p < 0.0001; Figure 2C’ and D’ see*), when compared to respective control Tat(−) mice treated with CBDA and CBDV, as indicated by the filled circles (Figure 2C,C’ and Figure 2D,D’ see*).

Sex differences in the analgesic response to minor phytocannabinoids were also observed. Tat(−) males treated with CBD displayed less visceral pain (p = 0.02; Figure 2E’ see‡) than did respective control Tat(−) males vehicle-treated, as indicated by the filled circles (Figure 2A’); however, this difference was not observed in Tat(−) females treated with CBD. Conversely, CBG significantly reduced writhes in Tat(+) females (p < 0.0001; Figure 2F see^) compared to Tat(+) vehicle-treated females, as indicated by the half-filled circles (Figure 2A), while CBG exacerbated Tat-mediated visceral pain in males (p < 0.0001; Figure 2F’ see∗). Notably, CBG was still efficacious in Tat(−) males (p = 0.04; Figure 2F’ see ‡) compared to their respective Tat(−) vehicle-treated controls, (Figure 2A’ see filled circles). CBN treatment also worsened visceral pain in Tat(+) females compared to Tat(−) females (p = 0.004; Figure 2G see*). However, such effect was not observed in Tat(+) males. Thus, sex, Tat, and minor phytocannabinoids exposure can modulate visceral pain.

### CBGA and CBG exert anti-nociceptive effects on male and female mice

We first assessed the baseline latencies of mice treated with both types of vehicle used (i.e. 0.9 % saline or a 1:1:18 mixture of EtOH/kolliphor/saline). No differences in baseline tail flick latency were observed among either sex in any vehicle group (Table 1). Minor phytocannabinoids, including CBGA, CBD, CBG, and CBN, that demonstrated efficacy for visceral pain were further assessed for their anti-nociceptive capacity in the tail flick assay. Efficacy and potency were evaluated using cumulative dose-response curves. Mice treated with oxycodone demonstrated greater anti-nociception across the five cumulative doses compared to any vehicle group in females [*F*(1,100) = 76.42, p < 0.05; Table1.S] and males [*F*(1,112) = 358.07, p < 0.05; Table1.S]. Accordingly, the acute anti-nociceptive effects of minor phytocannabinoids that were efficacious in the acetic acid writhing assay were then assessed for antinociception in the tail flick assay.

**Table 1:** Mean of tail withdrawal (s ± SEM) in adult male and female HIV-1 Tat-tg mice [Tat(+)] or their non-Tat expressing counterparts [Tat(−)] before and after vehicle injection.

Group	0.9 % Saline		Vehicle 1:1:18		Oxycodone	
	Tat(−) *n* = 8	Tat(+) *n* = 8	Tat(−) *n* = 8	Tat(+) *n* = 7	Tat(−) *n* = 8	Tat(+) *n* = 8
Females						
Baseline	1.7 ± 0.2	2.0 ± 0.2	1.7 ± 0.1	1.8 ± 0.2	1.7 ± 0.2	2.0 ± 0.2
Post-baseline	2.1 ± 0.1†	2.2 ± 0.3†	2.1 ± 0.1†	2.1 ± 0.2†	6.6 ± 0.1	8.0 ± 0.4
**Males**						
Baseline	2.2 ± 0.2	2.3 ± 0.1	1.9 ± 0.1	2.0 ± 0.1	1.4 ±0.1	1.9 ± 0.1
Post-baseline	2.3 ± 0.1†	2.3 ± 0.1†	2.7 ± 0.1†	2.7 ± 0.2†	7.5 ± 0.5	8.5 ± 0.3

†Indicates a significant interaction wherein indicated groups to differ from post-baseline oxycodone, p ≤ 0.05.

**Figure 3:**
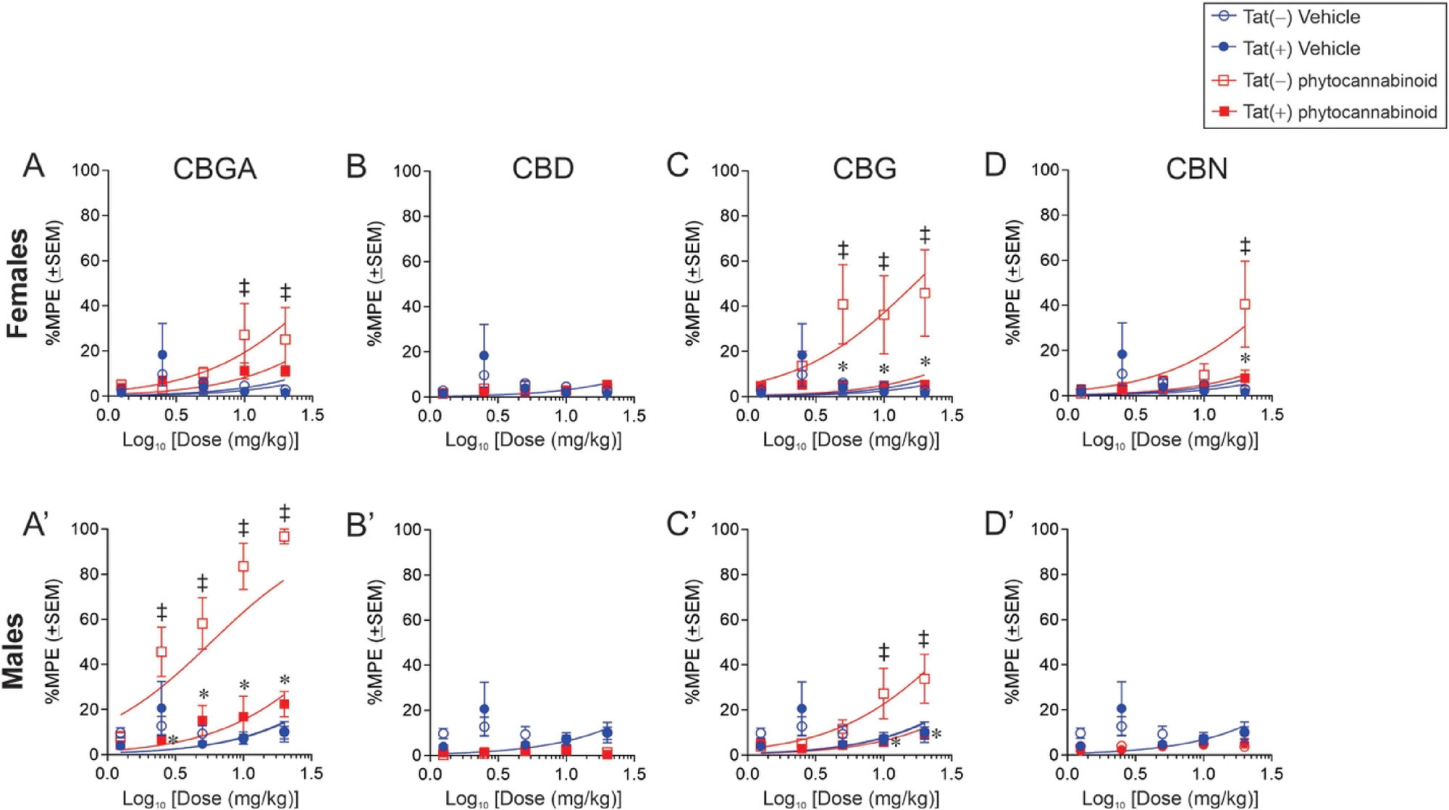
Anti-nociceptive capacity of minor phytocannabinoids in HIV-1 Tat-tg mice. Female and male mice (*n* = 6–12/group) that conditionally-expressed Tat [Tat(+)] or their non-Tat expressing counterparts [Tat(−)] were assessed at baseline and after a dose-response regimen of minor phytocannabinoids (1.25, 2.5, 5.0, 10.0, and 20.0) for the latency to warm-water tail withdrawal. Treatments included: (A,A′) CBGA, (B,B′) CBD, (C,C′) CBG, or (D,D′) CBN. *Indicates an interaction effect for Tat(+) mice to differ from control Tat(−) mice. ‡ indicates an interaction effect for Tat(−) mice to differ from their respective control Tat(−) vehicle-treated mice. *n* = 6–12/group, (two-way ANOVA repeated measurement, p ≤ 0.05).

Among females, Tat expression and drug condition significantly interacted across the five cumulative doses [*F*(24,329) = 2.13, p < 0.05; Figure 3] such that Tat(−) females treated with CBGA had greater anti-nociception at 10 and 20 mg/kg dosing compared to their respective vehicle (1:1:18)-treated Tat(−) controls (p = 0.03–0.05; Figure 3A see‡). Similar effects were seen for Tat(−) females treated with CBG displayed at 5, 10, and 20 mg/kg dosing (p = 0.0007–0.004; Figure 3C see‡) and for CBN at 20 mg/kg dosing (p = 0.02; Figure 3D see‡). However, Tat expression attenuated the analgesic capacity of CBG and CBN versus their counterpart Tat(−) females (p = 0.0006–0.04; Figure 3C and D see*). Irrespective of Tat genotype, we did not observe anti-nociceptive effects for CBD among females in this assay, indicating that its effects are selective to pain modality (Figure 3B).

Among male mice, Tat expression and drug condition also interacted to alter the anti-nociceptive response [*F*(24,412) = 2.51, p < 0.05; Figure 3]. Tat(−) males treated with CBGA (2.5, 5, 10, and 20 mg/kg; p *<* 0.0001*–*0.001; Figure 3A see‡) or those treated with CBG (10 and 20 mg/kg; p = 0.0001–0.0093; Figure 3 see‡) had greater anti-nociception compared to those Tat(−) mice treated with vehicle (1:1:18). However, Tat expression hindered the anti-nociceptive capacity of CBGA and CBG than did their control Tat(−) mice (p < 0.0001–0.005; Figure 3A, C see*). Neither CBD (Figure 3B) nor CBN (Figure 3D) exerted anti-nociceptive effects among male mice.

The potencies of the minor phytocannabinoids were assessed using the calculated half-maximal effective concentration (ED_50_) values. Significant interactions between Tat expression and drug condition were detected among females [*F*(9,395) = 12.89, p < 0.05; Table 2] and males [*F*(9,410) = 79.48, p < 0.05; Table 2]. In females, Tat expression reduced the potency for CBG and CBN compared to counterpart Tat(−) females (see* Table 2). Within males, Tat expression attenuated drug potency for CBGA and CBG compared to counterpart Tat(−) males (see* Table 2). Thus, Tat expression reduced the potency of minor phytocannabinoids in a sex-dependent manner.

**Table 2:** The calculated ED_50_ values (95 % confidence interval) of minor phytocannabinoids in adult HIV-1 Tat-tg mice [Tat(+)] or their non-Tat expressing counterparts [Tat(−)] (*n* = 7–9/group).

Treatment	Females		Males	
	Tat(−)	Tat(+)	Tat(−)	Tat(+)
CBGA	44.21 (25.26–98.19)	116.8 (84.18–181.00)	3.43 (2.362–4.928)	54.68 (36.08–94.69)*
CBD	343.60 (246.30–555.10)	306.40 (219.30–495.10)	753.00 (464.9–1898)	1,045.00 (531.70–16281)
CBG	15.91 (8.06–35.75)	237.80 (169.30–386.40)*	34.44 (22.87–56.86)	170.50 (125.10–258.7)*
CBN	43.51 (25.65–90.63)	212.30 (140.00–408.60)*	303.70 (202.70–577.10)	276.20 (200.20–434.30)

*Indicates a significant interaction effect for Tat(+) mice to differ from their counterpart Tat(−) mice, p ≤ 0.05.

## Discussion

The hypotheses that exposure to HIV-1 Tat would promote inflammatory and thermal pain that could be attenuated by minor phytocannabinoids were partly upheld. Tat exacerbated inflammatory visceral pain that was induced with acetic acid, but did not produce significant differences in baseline nociception associated with warm-water tail withdrawal. These data support prior findings that indicate some pain modalities (i.e. mechanical and thermal) to be exacerbated by Tat [19], 22]; whereas, effects on spinal cord-mediated anti-nociception are more modest [50] or are not observed [21], 51], 52]. Anti-nociceptive effects of minor phytocannabinoids were observed for both visceral and spinal-mediated nociception. CBGA and CBD attenuated Tat-mediated visceral pain in either sex, whereas CBG was efficacious in females and CBN was efficacious in males. In the warm water tail withdrawal assay, Tat significantly shifted the ED_50_ for CBG to the right in both sexes. A rightward shift was also observed for CBN in females and for CBGA in males. These findings are similar to those previously-observed for Tat to produce a rightward shift to opioids [20], 21], [51], [52], [53] and may indicate a more generalized desensitization to drugs that act on G-protein coupled receptors among other factors. Tat expression may reduce the potency of phytocannabinoids via receptor desensitization, manifesting as Tat(+) mice requiring higher doses of these compounds to achieve the same anti-nociceptive effects as their Tat(−) counterparts, thereby increasing abuse liability [20].

The mechanism(s) underlying HIV-related pain are poorly understood but are likely multifactorial. Neuroinflammation is believed to contribute to the development of chronic pain indirectly [54], 55]. In support, neurotoxic Tat protein is still detected within the CSF of virally-suppressed PLWH [17], 18], 56]. Tat promotes neuroinflammation [57] and excitotoxicity within the CNS [58]. In addition, Tat expression may dysregulate pain transduction in Tat-transgenic mice via reduction in nerve fiber density [22], 23] and sensory nerve action potentials [22]. Subsequently, Tat alters sensory afferent fibers (unmyelinated C- and thinly myelinated Aδ-fibers) which is expected to change the excitatory-inhibitory homeostasis within the spinal dorsal horn [59]. These fibers transmit information from thermoreceptors (TRPV1, TRPV2, TRPV3, TRPV4, and TRM8) and CB receptors. Abnormalities in anti-nociceptors are linked to neuropathic pain. For example, an upregulation of transient receptor potential vanilloid-1 (TRPV-1) channels has been observed in medium and large injured neurons within the DRG [60]. Peripheral nerve inflammation has also been shown to increase TRPV1 protein levels, though related mRNA expression remains unaffected in the DRG [61], TRPV1 expression is upregulated in inflamed hindpaws [62]. Furthermore, the expression of CB2 receptors is enhanced in both the spinal cord and DRG during chronic inflammatory pain as well as in spinal nerve injury models using rats [63]. Such CB2 upregulation is also observed on microglia during chemotherapy-induced neuropathic pain, peripheral nerve injury [64], 65], and in the brains of simian immunodeficiency virus-infected macaques [66]. In the latter, CB2 upregulation is particularly observed on perivascular macrophages, nodular microglia, and CD8^+^ T-lymphocytes [66], 67]. These findings collectively support the involvement of neuroinflammatory signaling in neuropathic and HIV-related pain states. The present data are consistent with dysregulation of sensory afferent neurons and spinal ascending pathways and identify aspects that may be modified by Tat [68]. The modest effect of Tat on the baseline tail flick reflex could be due to actions on unmyelinated C-fibers. In support, previous studies report capsaicin-induced C-fiber neurotoxicity [69], 70] to coincide with a reduced tail flick response [69]. In PLWH, these effects may be driven by ‘entourage’ effects of HIV proteins. Combined gp120 and Tat produce a significant reduction in the mean axonal length in cultured human dorsal root ganglia [14]. While the present worked is focused on Tat, these findings align with clinical observations [71], [72], [73] that demonstrate cannabis to reduce HIV- related pain symptomology.

The pharmacological action(s) of cannabis are not fully understood. However, it is known that cannabis exerts an immunomodulatory capacity. In PLWH, cannabis consumption suppresses immune activation, including lowering the frequency of activated T cells (CD4^+^ and CD8^+^) [74], activated monocytes (CD16^+^), and interferon-γ-inducible protein 10 [75]. Cannabis also lowered pro-inflammatory markers in the CSF [76] and plasma [77] of PLWH, leading to improved cognitive performance [76]. The specific effects of cannabis constituents have been assessed in cultured cells. CBD reduces the secretion of pro-apoptotic caspase-1 gene expression in the latently-infected microglial cells (HC69.5) [78]. However, the cytoprotective effects of CBD are complex as it is also observed to induce autophagy in human neuroblastoma SH-SY5Y cells, a murine astrocyte cell line [79], and HIV-infected human microglia [72]. While the mechanisms are still being investigated, CBD is found to attenuate activation of CB receptors and TRPV-1 channels [71], resulting in an attenuation of Type I IFN responses in HIV-infected human microglia [80]. Furthermore, CBD, CBG, CBDA, CBGA, and CBN are shown to stimulate and desensitize TRPV-1 channels in HEK-293 cells [81], 82] resulting in potentially paradoxical effects for analgesia [82]. In addition to TRPV-1 desensitization, CBN and CBG also antagonized TRPM8 (melastatin) channels [81] thereby altering cold pain sensation [83]. Herein, we find CBGA, CBD, and CBN to attenuate Tat-mediated visceral pain which is expected to be largely driven by pro-inflammatory factors. CBGA and CBG also ameliorated the tail flick reflex in Tat(−) mice; however, not in Tat(+) mice. It is possible that Tat impairs the latter modality through mechanisms beyond inflammation that are not ameliorated by the minor phytocannabinoids assessed. Surprisingly, we also find stark sex differences in the efficacy of these cannabinoids. CBG ameliorated visceral pain in Tat(+) females; however, it exacerbated the pain in counterpart Tat(+) males. CBN was analgesic in Tat(−) females but not in Tat(−) males. Thus, cannabis-based therapy might mitigate HIV-induced neuroinflammation and its consequences of chronic pain among PLWH. However, there may be sex differences in the efficacy of the anti-nociceptive response.

Preclinical studies have investigated sex differences in the physiological effects of cannabis [84]. Female rats treated with cannabinoid agonists exhibited a greater anti-nociceptive response in the tail flick test and mechanical paw pressure tasks compared to males [85], 86]. Additionally, female rats were found to be more sensitive than males to low doses of Δ^9^-THC characterized by increased wheel running [87]. Although these differences are poorly understood, hormonal and pharmacokinetic factors may contribute [88]. In gonadectomized female rats, estradiol treatment enhanced Δ^9^-THC’s analgesic effects [89] and attenuated inflammatory pain in the formalin test [90]. Estradiol exerts its own anti-nociceptive capacity which may be mediated in part by TRPV-1 and thus could exert additive effects similar to cannabinoids [91]. Moreover, female rats also display higher content of Δ^9^-THC in the brain compared to males [86] concurrent with greater CB1 expression [92]. Hormonal changes during the estrous cycle have not been systematically assessed in this context but may influence the physiological response to the anti-HIV efficacy of minor phytocannabinoids.

Overall, PLWH are more vulnerable to the development of chronic pain, resulting in physical disability and a reduced quality of life. The current pharmacological treatments for managing HIV-related pain lack efficacy and are associated with the risk of substance abuse. The medicinal use of non-psychoactive cannabis constituents for pain management might greatly benefit this population which is at a greater risk for opioid addiction and substance abuse [93], 94]. Further study is warranted to evaluate cannabinoid-based therapeutics for their potential sex-dependent effects.

## Supplementary Material

Supplementary Material Details
