# Supplementary material for: Sex differences in the capacity of minor phytocannabinoids to attenuate nociceptive insults in HIV-1 Tat-expressing mice
Source: NeuroImmune Pharm Ther. 2025 Jul 17;4(3):303–13. doi: 10.1515/nipt-2024-0025 (PMC12601221; doi:10.1515/nipt-2024-0025)
Supplement: Supplementary file 1 — Supplementary Material Details [file j_nipt-2024-0025_suppl_001.docx]

**Table 1S.** Cumulative dose-response (± SEM) in adult male and female HIV-1 Tat-tg mice [Tat(+)] or their non-Tat expressing counterparts [Tat(−)] for the antinociceptive effects of vehicle (1:1:18) and oxycodone.

|  | **Vehicle (1:1:18)** | | | | |
| --- | --- | --- | --- | --- | --- |
|  | **1.25 mg/kg** | **2.5 mg/kg** | **5 mg/kg** | **10 mg/kg** | **20 mg/kg** |
| Female Tat(−) | 3.0±1.2 | 10±1.6 | 6.2±1.2 | 4.7±1.8 | 3.0±1.2 |
| Female Tat(+) | 1.5±0.9 | 5.8.5±3.8 | 5.5±4.5 | 2.4±0.8 | 2.1±1.6 |
|  |  |  |  |  |  |
| Male Tat(−) | 3.0±1.2 | 13 ±4.2 | 9.4±3.6 | 7.3±2.7 | 10±4.5 |
| Male Tat(+) | 4.4±1.7 | 9.3±3.8 | 5.5±1.6 | 8.0±2.1 | 9.2±3.1 |
|  |  |  |  |  |  |
|  | **Oxycodone** | | | | |
|  |  | | | | |
| Female Tat(−) | 13±2.7^†^ | 63±15^†^ | 70±15^†^ | 68±15^†^ | 79±15^†^ |
| Female Tat(+) | 38±17^†^ | 81±13^†^ | 88±12^†^ | 100±0.0^†^ | 100±0.0^†^ |
|  |  |  |  |  |  |
| Male Tat(−) | 15±3.9^†^ | 59±13^†^ | 82±12^†^ | 100±0.0^†^ | 100±0.0^†^ |
| Male Tat(+) | 44±12^†^ | 63±14^†^ | 100±0.0^†^ | 100±0.0^†^ | 100±0.0^†^ |

† Indicates a main effect wherein indicated groups to differ from vehicle-treated groups.
